# Association of Obstructive Sleep Apnea With the Risk of Male Infertility in Taiwan

**DOI:** 10.1001/jamanetworkopen.2020.31846

**Published:** 2021-01-21

**Authors:** Yi-Han Jhuang, Chi-Hsiang Chung, I-Duo Wang, Chung-Kan Peng, En Meng, Wu-Chien Chien, Ping-Ying Chang

**Affiliations:** 1 Department of Surgery, Tri-Service General Hospital, National Defense Medical Center, Taipei, Taiwan; 2 School of Public Health, National Defense Medical Center, Taipei, Taiwan; 3 Department of Medical Research, Tri-Service General Hospital, National Defense Medical Center, Taipei, Taiwan; 4 Taiwanese Injury Prevention and Safety Promotion Association, Taipei, Taiwan; 5 Division of Pulmonary and Critical Care Medicine, Department of Internal Medicine, Tri-Service General Hospital, National Defense Medical Center, Taipei, Taiwan; 6 Division of Urology, Department of Surgery, Tri-Service General Hospital, National Defense Medical Center, Taipei, Taiwan; 7 Graduate Institute of Life Sciences, National Defense Medical Center, Taipei, Taiwan; 8 Division of Hematology/Oncology, Department of Internal Medicine, Tri-Service General Hospital, National Defense Medical Center, Taipei, Taiwan

## Abstract

**Importance:**

Obstructive sleep apnea (OSA) has been proposed as a risk factor in infertility. However, to date, the association between OSA and male infertility has not been examined in a population-based study.

**Objective:**

To investigate the risk factor of OSA in male infertility and the outcome of OSA treatment for the risk of male infertility.

**Design, Setting, and Participants:**

This case-control population-based study collected data from the Longitudinal Health Insurance Database, a subset of the National Health Insurance Research Database in Taiwan. Male patients with a diagnosis of infertility and at least 3 outpatient visits or 1 hospitalization between January 1, 2000, and December 31, 2013, were included and matched by age, sex, and date of infertility diagnosis with individuals without an infertility diagnosis. Data analysis was performed from October 22, 2018, to April 22, 2019.

**Exposures:**

Patients with male infertility and randomly selected patients without male infertility were matched using a 1:4 propensity score matching ratio.

**Main Outcomes and Measures:**

A primary outcome was the risk factor of OSA (diagnosed through polysomnography). A secondary outcome was the association of the risk of male infertility with OSA exposure time interval (short term, middle term, and long term) and OSA management (ie, none, continuous positive airway pressure, uvulopalatopharyngoplasty, or both).

**Results:**

A total of 4607 male patients with infertility (mean [SD] age, 34.18 [5.44] years) and 18 428 control patients (mean [SD] age, 34.28 [5.81] years) were included. In the multivariate conditional logistic regression analysis, OSA was an independent risk factor associated with infertility (adjusted odds ratio [OR], 1.24; 95% CI, 1.10-1.64; *P* = .003). The absolute risk was 0.204 (95% CI, 0.092-0.391). For patients with OSA in the group without treatment, the adjusted OR was 1.80 (95% CI, 1.56-2.07; *P* < .001) for infertility compared with patients without OSA.

**Conclusions and Relevance:**

Results of this study support the hypothesis that OSA increases the risk of infertility in male patients, and the risk is associated with the OSA exposure time. Furthermore, no OSA management or treatment is associated with a higher infertility risk.

## Key Points

**Question:**

What is the association between obstructive sleep apnea and male infertility?

**Findings:**

In this large case-control study of 4607 men with infertility and 18 428 control patients, obstructive sleep apnea was associated with a 1.24-fold higher risk of developing infertility.

**Meaning:**

Findings from this study suggest that sleep disturbance, including obstructive sleep apnea, has an association with metabolic and neurocognitive functions and that obstructive sleep apnea increases the risk of infertility in men.

## Teaser

This case-control study examines the demographic variables, comorbidities, exposure to obstructive sleep apnea, and obstructive sleep apnea therapies in adult men with or without an infertility diagnosis.

## Introduction

Infertility is typically defined as a failure to conceive with unprotected sex after at least 12 months.^1^ A previous study reported that the 12-month prevalence of infertility was 3.5% to 16.7% in higher-income nations and 6.9% to 9.3% in lower-income nations. The estimated overall median prevalence was 9%.^2^ In Taiwan, the prevalence of infertility was estimated to be 10% to 15% according to the National Health Insurance Research Database (NHIRD).^3^ Levine et al^4^ conducted a systematic review and meta-regression analysis that presented evidence of a decline in sperm quality over the past decades worldwide. The male factor was estimated to be present in about 50% of infertility cases.^5^

Although approximately 50% of patients in 1 study were labeled as having idiopathic male infertility, the known etiologic factors were congenital and acquired.^6^ Obesity and metabolic syndrome have been recognized as factors in idiopathic male infertility.^7,8^ Obstructive sleep apnea (OSA) has been proposed as a modulating factor in obesity and infertility.^9,10^ The severity of OSA also correlated with the reproductive hormone after adjustment for obesity.^10,11^ The association between OSA and testosterone has been studied previously.^11,12,13^ The association of OSA therapy with reproductive hormones has also been investigated, but conflicting results were found.^13,14,15^ However, to date, the association between OSA and male infertility has not been examined in a population-based study. Thus, using a large data set available in Taiwan, we conducted a nationwide nested case-control study to investigate the hypothesis that OSA is a risk factor in male infertility.

## Methods

The institutional review board of the Tri-Service General Hospital approved this study and waived the requirement of individual written consent for the purpose of medical research and anonymous analysis (TSGHIRB No. B-109-09). All analyses were performed according to the Declaration of Helsinki,^16^ and reporting followed the Strengthening the Reporting of Observational Studies in Epidemiology (STROBE) guideline.^17^

### Data Sources, Study Design, and Population

This study collected data from the Longitudinal Health Insurance Database, a subset of the NHIRD, that are representative of the entire population of Taiwan.^18,19^ This registry contains wide-ranging data from the National Health Insurance system in Taiwan, which was introduced in 1995 to provide comprehensive population coverage, including demographic characteristics, clinical visiting times, and disease diagnoses. The *International Classification of Diseases, Ninth Revision, Clinical Modification* (*ICD-9-CM*) diagnosis codes are used in the National Health Insurance program. 

Male patients with a diagnosis of infertility (*ICD-9-CM* code 606) between January 1, 2000, and December 31, 2013, were included in the study. However, patients with azoospermia, erectile dysfunction, early ejaculation, and infertility with extratesticular causes were excluded. The *ICD-9-CM* codes that we used are summarized in eTable 1 in the Supplement. The index date was defined as the date of infertility diagnosis. The specific exclusion criteria were age younger than 18 years; a history of underlying drug exposure, including alkylating agents, platinum, and antiepileptic medications; and a diagnosis of any of the following conditions before the index date: (1) infertility; (2) genital warts, chlamydia, gonorrhea, syphilis, and HIV; (3) epididymitis, orchitis, and mumps orchitis; (4) varicocele; (5) genital tuberculosis; (6) alcohol abuse or dependence syndrome and drug abuse or dependence; and (7) endocrine disorders, hypogonadotropic hypogonadism, cryptorchidism, testicular hypofunction, adrenogenital disorders, and thyroid disorders. We randomly selected individuals without an infertility diagnosis as the control group, and they were matched with the patient group by age, sex, and index date using a 4-fold propensity score matching. Among these control patients, those younger than 18 years and with a medical history as indicated in the exclusion criteria were excluded. The flowchart of patients is shown in eFigure 1 in the Supplement.

A primary outcome was the risk factor of OSA. Patients with OSA were diagnosed after polysomnography and had at least 3 outpatient visits or 1 hospitalization according to the medical records between January 1, 2000, and December 31, 2013. In Taiwan, continuous positive airway pressure (CPAP) is a suggested treatment for patients with 1 of the following indications: apnea-hypopnea index higher than 40, respiratory disturbance index of 40 or higher, or persistent severe oxygen desaturation (≤85%) for more than 1 hour.

A secondary outcome was the association of OSA treatment with the risk of male infertility. The patients with OSA were divided into 4 treatment groups: (1) without management, (2) with uvulopalatopharyngoplasty (UPPP), (3) with CPAP, and (4) with both UPPP and CPAP. The OSA exposure time interval to infertility diagnosis was divided into less than 1 year (short-term interval), 1 to less than 5 years (middle-term interval), or 5 or more years (long-term interval). These exposure time intervals represent the association of OSA with male infertility.

### Propensity Score Matching and Comorbidities

We used 1:4 propensity score matching to reduce the selection bias. The propensity score was calculated with a multivariate conditional logistic regression analysis to estimate the probability of treatment assignment according to baseline variables and comorbidities, including age, obesity, diabetes, hyperlipidemia, chronic kidney disease, hypertension, coronary artery disease, congestive heart failure, chronic obstructive pulmonary disease, cerebrovascular accident, hepatitis B virus, hepatitis C virus, liver cirrhosis, epilepsy, cancer, anxiety disorders, and depressive disorders. This approach is in accordance with that used in a previous study.^20^

### Statistical Analysis

All statistical analyses were performed from October 22, 2018, to April 22, 2019, using IBM SPSS Statistics, version 22 (IBM Corp). We used a 2-tailed, unpaired *t* test to analyze continuous data and a χ^2^ test to analyze categorical data for baseline characteristics. Continuous data were presented as means with SD, whereas categorical data were presented as frequencies with percentages for baseline characteristics. The multivariate conditional logistic regression model was used to investigate the odds ratio (OR) with 95% CI of patients with OSA for developing male infertility after adjusting for demographic data and medical comorbidities. Two-sided *P* < .05 was considered to indicate statistical significance.

## Results

A total of 4607 patients (mean [SD] age, 34.18 [5.44] years) with a diagnosis of male infertility (*ICD-9-CM* code 606.x) were included in this study. A matched group of 18 428 patients (mean [SD] age, 34.28 [5.81] years) was randomly selected as the control group (eFigure 1 in the Supplement). Table 1 describes the baseline characteristics of participants with or without male infertility. The substantial differences in the prevalence of hypertension, diabetes, hyperlipidemia, chronic obstructive pulmonary disease, chronic kidney disease, coronary artery disease, stroke, obesity, anxiety, and depression are noted between the study and control groups. For example, compared with the control group, the study group had a higher prevalence of OSA (3.17 vs 2.59; *P* = .02).

**Table 1.** Baseline Characteristics in Participants With or Without Male Infertility

Variable	No. (%)			*P* value^**a**^
	Total	Study group	Control group	
Total	23 035 (100)	4607 (20)	18 428 (80)	
Age, mean (SD), y	34.26 (5.74)	34.18 (5.44)	34.28 (5.81)	.31
Age group, y				
18-24	725 (3.2)	145 (3.2)	580 (3.2)	>.99
25-29	4315 (18.7)	863 (18.7)	3452 (18.7)	
30-34	8640 (37.5)	1728 (37.5)	6912 (37.5)	
35-39	6130 (26.6)	1226 (26.6)	4904 (26.6)	
40-44	2345 (10.2)	469 (10.2)	1876 (10.2)	
≥45	880 (3.8)	176 (3.8)	704 (3.8)	
OSA				
Without	22 424 (97.4)	4461 (96.8)	17 963 (97.5)	.02
With	611 (2.7)	146 (3.2)	465 (2.5)	
Hypertension				
Without	21 170 (91.9)	4167 (90.5)	17 003 (92.3)	<.001
With	1865 (8.1)	440 (9.6)	1425 (7.7)	
Diabetes				
Without	21 746 (94.4)	4292 (93.2)	17 454 (94.7)	<.001
With	1289 (5.6)	315 (6.8)	974 (5.3)	
Hyperlipidemia				
Without	22 277 (96.7)	4411 (95.8)	17 866 (97.0)	<.001
With	758 (3.3)	196 (4.3)	562 (3.1)	
COPD				
Without	22 594 (98.1)	4431 (96.2)	18 163 (98.6)	<.001
With	441 (1.9)	176 (3.8)	265 (1.4)	
CKD				
Without	22 633 (98.3)	4495 (97.6)	18 138 (98.4)	<.001
With	402 (1.8)	112 (2.4)	290 (1.6)	
CAD				
Without	22 381 (97.2)	4445 (96.5)	17 936 (97.3)	.002
With	654 (2.8)	162 (3.5)	492 (2.7)	
CHF				
Without	22 832 (99.1)	4563 (99.0)	18 269 (99.1)	.55
With	203 (0.9)	44 (1.0)	159 (0.9)	
Liver cirrhosis				
Without	22 502 (97.7)	4504 (97.8)	17 998 (97.7)	.69
With	533 (2.3)	103 (2.2)	430 (2.3)	
Stroke				
Without	22 612 (98.2)	4471 (97.1)	18 141 (98.4)	<.001
With	423 (1.8)	136 (3.0)	287 (1.6)	
Cancer				
Without	21 566 (93.6)	4315 (93.7)	17 251 (93.6)	.90
With	1469 (6.4)	292 (6.3)	1177 (6.4)	
Obesity				
Without	22 870 (99.3)	4548 (98.7)	18 322 (99.4)	<.001
With	165 (0.7)	59 (1.3)	106 (0.6)	
Epilepsy				
Without	22 816 (99.1)	4563 (99.0)	18 253 (99.1)	.97
With	219 (1.0)	44 (1.0)	175 (1.0)	
HBV				
Without	22 343 (97.0)	4466 (96.9)	17 877 (97.0)	.80
With	692 (3.0)	141 (3.1)	551 (3.0)	
HCV				
Without	22 854 (99.2)	4565 (99.1)	18 289 (99.3)	.28
With	181 (0.8)	42 (0.9)	139 (0.8)	
Anxiety				
Without	22 818 (99.1)	4510 (97.9)	18 308 (99.4)	<.001
With	217 (0.9)	97 (2.1)	120 (0.7)	
Depression				
Without	22 694 (98.5)	4482 (97.3)	18 212 (98.8)	<.001
With	341 (1.5)	125 (2.7)	216 (1.2)	

Abbreviations: CAD, coronary artery disease; CHF, congestive heart failure; CKD, chronic kidney disease; COPD, chronic obstructive pulmonary disease; HBV, hepatitis B virus; HCV, hepatitis C virus; OSA, obstructive sleep apnea.

^a^ *P* values were calculated using χ^2^ or Fisher exact test for categorical variables and *t* test for continuous variables.

In the multivariate conditional logistic regression analysis, a significantly higher risk of infertility was observed in patients with comorbidities, including hypertension (adjusted OR, 1.40; 95% CI, 1.09-2.70; *P* = .001), diabetes (adjusted OR, 2.01; 95% CI, 1.21-3.15; *P* < .001), hyperlipidemia (adjusted OR, 1.80; 95% CI, 1.25-2.80; *P* < .001), chronic obstructive pulmonary disease (adjusted OR, 2.13; 95% CI, 1.14-3.30; *P* < .001), chronic kidney disease (adjusted OR, 1.70; 95% CI, 1.01-2.76; *P* = .04), coronary artery disease (adjusted OR, 1.56; 95% CI, 1.01-2.13; *P* = .04), liver cirrhosis (adjusted OR, 1.56; 95% CI, 1.09-1.99; *P* = .001), obesity (adjusted OR, 3.01; 95% CI, 1.76-8.34; *P* < .001), anxiety (adjusted OR, 1.99; 95% CI, 1.10-3.00; *P* < .001), and depression (adjusted OR, 2.16; 95% CI, 1.34-3.02; *P* < .001). Patients younger than 40 years had a statistically significantly higher risk of infertility (eg, 18-24 years adjusted OR, 2.86; 95% CI, 1.87-4.26; *P* < .001; 40-44 years adjusted OR, 1.09; 95% CI, 0.97-1.50; *P* = .16) than those aged 45 years or older (reference). Obstructive sleep apnea appeared to be an independent risk factor in infertility (adjusted OR, 1.24; 95% CI, 1.10-1.64; *P* = .003). The absolute risk was 0.204 (95% CI, 0.092-0.391). These findings are shown in Table 2.

**Table 2.** Factors of Infertility

Variable	Crude OR (95% CI)	*P* value^**a**^	Adjusted OR (95% CI)	*P* value^**a**^
OSA				
Without	1 [Reference]		1 [Reference]	
With	1.26 (1.16-1.69)	.001	1.24 (1.10-1.64)	.003
Age group, y				
18-24	3.06 (1.90-5.98)	<.001	2.86 (1.87-4.26)	<.001
25-29	1.45 (1.10-2.05)	<.001	1.60 (1.10-1.90)	<.001
30-34	1.19 (1.00-1.98)	.045	1.57 (1.10-1.86)	.002
35-39	1.09 (0.98-1.34)	.17	1.13 (1.00-1.57)	.049
40-44	1.01 (0.88-1.21)	.22	1.09 (0.97-1.50)	.16
≥45	1 [Reference]		1 [Reference]	
Hypertension				
Without	1 [Reference]		1 [Reference]	
With	1.54 (1.15-2.98)	<.001	1.40 (1.09-2.70)	.001
Diabetes				
Without	1 [Reference]		1 [Reference]	
With	2.16 (1.45-3.01)	<.001	2.01 (1.21-3.15)	<.001
Hyperlipidemia				
Without	1 [Reference]		1 [Reference]	
With	1.91 (1.31-2.90)	<.001	1.80 (1.25-2.80)	<.001
COPD				
Without	1 [Reference]		1 [Reference]	
With	2.54 (1.60-3.80)	<.001	2.13 (1.14-3.30)	<.001
CKD				
Without	1 [Reference]		1 [Reference]	
With	2.11 (1.47-3.15)	<.001	1.70 (1.01-2.76)	.04
CAD				
Without	1 [Reference]		1 [Reference]	
With	2.14 (1.24-2.99)	<.001	1.56 (1.01-2.13)	.04
CHF				
Without	1 [Reference]		1 [Reference]	
With	1.09 (0.72-1.85)	.36	1.01 (0.42-1.56)	.67
Liver cirrhosis				
Without	1 [Reference]		1 [Reference]	
With	1.35 (1.01-1.57)	.04	1.56 (1.09-1.99)	.001
Stroke				
Without	1 [Reference]		1 [Reference]	
With	1.01 (0.60-1.77)	.49	1.12 (0.99-1.96)	.06
Cancer				
Without	1 [Reference]		1 [Reference]	
With	1.16 (1.00-1.76)	.053	0.95 (0.70-1.42)	.18
Obesity				
Without	1 [Reference]		1 [Reference]	
With	4.49 (2.45-9.80)	<.001	3.01 (1.76-8.34)	<.001
Epilepsy				
Without	1 [Reference]		1 [Reference]	
With	0.00	>.99	0.00	>.99
HBV				
Without	1 [Reference]		1 [Reference]	
With	1.15 (0.74-1.68)	.24	1.24 (0.89-1.86)	.12
HCV				
Without	1 [Reference]		1 [Reference]	
With	1.35 (1.06-1.80)	.002	1.23 (0.91-1.75)	.33
Anxiety				
Without	1 [Reference]		1 [Reference]	
With	3.72 (1.69-5.13)	<.001	1.99 (1.10-3.00)	<.001
Depression				
Without	1 [Reference]		1 [Reference]	
With	3.98 (1.88-5.98)	<.001	2.16 (1.34-3.02)	<.001

Abbreviations: CAD, coronary artery disease; CHF, congestive heart failure; CKD, chronic kidney disease; COPD, chronic obstructive pulmonary disease; HBV, hepatitis B virus; HCV, hepatitis C virus; OR, odds ratio; OSA, obstructive sleep apnea.

^a^ *P* values were calculated using χ^2^ or Fisher exact test for categorical variables and *t* test for continuous variables.

We used a stratified logistic regression analysis to evaluate the association of OSA with each variable. Obstructive sleep apnea appeared to be associated with an increased risk of infertility in all 13 age groups, except in those aged 40 years or older (adjusted OR, 1.09; 95% CI, 0.97-1.44; *P* = .12). Men with OSA and comorbidities had a statistically significantly increased risk of infertility (Table 3). For example, among patients with OSA and infertility, compared with those without infertility, hypertension was a statistically significant risk factor (adjusted OR, 1.73; 95% CI, 1.45-2.30; *P* < .001). Furthermore, we analyzed the joint outcome of OSA, obesity, and cardiometabolic disease. The association between OSA and male infertility was statistically significant in patients without obesity (adjusted OR, 2.94; 95% CI, 2.10-6.11; *P* < .001) or cardiometabolic diseases (adjusted OR, 1.89; 95% CI, 1.30-2.89; *P* < .001) after adjustment (eFigures 2 and 3 in the Supplement).

**Table 3.** Factors of Infertility Stratified by Variables

Variable	Infertility				Adjusted OR (95% CI)	*P* value^**a**^
	With		Without			
	OSA, No. (%)	Population	OSA, No. (%)	Population		
Overall	146 (3.2)	4607	465 (0.7)	18 428	1.24 (1.10-1.64)	.003
Age group, y						
18-24	5 (3.5)	145	6 (57.5)	580	3.29 (2.93-4.36)	<.001
25-29	25 (2.9)	863	80 (3.6)	3452	1.23 (1.10-1.94)	.001
30-34	58 (3.4)	1728	179 (1.9)	6912	1.28 (1.14-1.85)	<.001
35-39	39 (3.2)	1226	131 (2.4)	4904	1.17 (1.05-1.56)	.03
40-44	16 (3.4)	469	58 (5.9)	1876	1.09 (0.97-1.44)	.12
≥45	3 (1.7)	176	11 (15.5)	704	1.08 (0.95-1.40)	.25
Hypertension						
Without	113 (2.7)	4167	404 (0.7)	17 003	1.13 (1.00-1.49)	.04
With	33 (7.5)	440	61 (12.3)	1425	1.73 (1.45-2.30)	<.001
Diabetes						
Without	125 (2.9)	4292	439 (0.7)	17 454	1.14 (1.02-1.56)	.03
With	21 (6.7)	315	26 (25.6)	974	2.46 (2.11-3.27)	<.001
Hyperlipidemia						
Without	128 (2.9)	4411	442 (0.7)	17 866	1.16 (1.03-1.53)	.04
With	18 (9.2)	196	23 (39.9)	562	2.12 (1.88-3.03)	<.001
COPD						
Without	125 (2.8)	4431	454 (0.6)	18 163	1.23 (1.03-1.69)	.02
With	21 (11.9)	176	11 (108.5)	265	2.84 (2.02-4.99)	<.001
CKD						
Without	139 (3.1)	4495	458 (0.7)	18 138	1.21 (1.08-1.60)	.008
With	7 (6.3)	112	7 (89.3)	290	2.55 (1.99-3.88)	<.001
CAD						
Without	131 (3.0)	4445	447 (0.7)	17 936	1.17 (1.04-1.53)	.02
With	15 (9.3)	162	18 (51.4)	492	2.5 (1.99-3.40)	<.001
CHF						
Without	143 (3.2)	4563	457 (0.7)	18 269	1.24 (1.12-1.69)	<.001
With	3 (6.8)	44	8 (85.2)	159	1.34 (1.19-1.70)	<.001
Liver cirrhosis						
Without	144 (3.2)	4504	460 (0.7)	17 998	1.23 (1.10-1.70)	.001
With	2 (1.9)	103	5 (38.8)	430	1.65 (1.47-2.05)	<.001
Stroke						
Without	143 (3.2)	4471	460 (0.7)	18 141	1.24 (1.11-1.69)	<.001
With	3 (2.2)	136	5 (44.1)	287	1.25 (1.11-1.70)	<.001
Cancer						
Without	141 (3.3)	4315	451 (0.7)	17 251	1.23 (1.10-1.64)	.02
With	5 (1.7)	292	14 (12.2)	1177	1.42 (1.24-1.87)	<.001
Obesity						
Without	120 (2.6)	4548	447 (0.6)	18 322	1.07 (0.95-1.47)	.27
With	26 (44.1)	59	18 (244.8)	106	2.96 (1.27-6.13)	<.001
Epilepsy						
Without	146 (3.2)	4563	465 (0.7)	18 253	1.24 (1.10-1.64)	.003
With	0 (0)	44	0 (0)	175	NA	NA
HBV						
Without	143 (3.2)	4466	458 (0.7)	17 877	1.23 (1.10-1.64)	.007
With	3 (2.1)	141	7 (30.4)	551	1.65 (1.47-2.19)	<.001
HCV						
Without	145 (3.2)	4565	463 (0.7)	18 289	1.24 (1.10-1.63)	.006
With	1 (2.4)	42	2 (119.1)	139	1.63 (1.43-2.19)	<.001
Anxiety						
Without	142 (3.2)	4510	463 (0.7)	18 308	1.23 (1.06-1.63)	.02
With	4 (4.1)	97	2 (206.2)	120	2.44 (1.57-3.98)	<.001
Depression						
Without	138 (3.1)	4482	460 (2.5)	18 212	1.2 (1.00-1.60)	.048
With	8 (6.4)	125	5 (2.3)	216	2.77 (1.41-3.91)	<.001

Abbreviations: CAD, coronary artery disease; CHF, congestive heart failure; CKD, chronic kidney disease; COPD, chronic obstructive pulmonary disease; HBV, hepatitis B virus; HCV, hepatitis C virus; NA, not applicable; OR, odds ratio; OSA, obstructive sleep apnea.

^a^ *P* values were calculated using χ^2^ or Fisher exact test for categorical variables and *t* test for continuous variables.

We divided the OSA exposure time interval into less than 1 year (short term), 1 to less than 5 years (middle term), and 5 or more years (long term) before infertility diagnosis. We found a pattern of an increasing risk of infertility associated with the increasing exposure time interval of OSA (Table 4). In the exposure time interval of more than 5 years, the risk of infertility was higher in patients with OSA than in those without (adjusted OR, 1.86; 95% CI, 1.23-2.21; *P* < .001).

**Table 4.** Obstructive Sleep Apnea (OSA) Exposure Period Before Infertility Diagnosis

Exposure time interval	With OSA vs without OSA [Reference]	
	Adjusted OR (95% CI)^**a**^	*P* value
Overall	1.24 (1.10-1.64)	.003
<1 y	1.17 (1.08-1.57)	.02
≥1 to <5 y	1.26 (1.12-1.67)	<.001
≥5 y	1.86 (1.23-2.21)	<.001

Abbreviation: OR, odds ratio.

^a^ ORs adjusted for the variables listed in Table 2.

We evaluated the association of OSA management, including UPPP and CPAP treatment, with the subsequent development of infertility. Approximately 50% of the patients with OSA received OSA treatment in the group with infertility (n = 60) and the group without infertility (n = 246) (Table 5). The patients with OSA who did not receive any treatment had a statistically significantly higher risk of infertility (adjusted OR, 1.80; 95% CI, 1.56-2.07; *P* < .001).

**Table 5.** Factors of Infertility Stratified by Treatment Type

Treatment group	OSA, No. (%)		With OSA vs without OSA [Reference]	
	With infertility (n = 4607)	Without infertility (n = 18 428)	Adjusted OR (95% CI)	*P* value
Overall	146 (3.2)	465 (2.5)	1.24 (1.10-1.64)	.003
Without management	86 (1.9)	219 (1.2)	1.80 (1.56-2.07)	<.001
CPAP only	37 (0.8)	149 (0.8)	0.99 (0.86-1.20)	.14
UPPP only	20 (0.4)	83 (0.5)	0.98 (0.84-1.13)	.27
CPAP and UPPP	3 (0.1)	14 (0.1)	0.98 (0.84-1.10)	.39

Abbreviations: CPAP, continuous positive airway pressure; OR, odds ratio; OSA, obstructive sleep apnea; UPPP, uvulopalatopharyngoplasty.

## Discussion

Obstructive sleep apnea is a common sleep disorder caused by repetitive pharyngeal collapse during sleep. This case-control study demonstrated that patients with OSA had a 1.24-fold greater risk of infertility than the control group. Young age (<40 years) and other comorbidities, including diabetes, hypertension, hyperlipidemia, chronic obstructive pulmonary disease, coronary artery disease, chronic kidney disease, liver cirrhosis, obesity, anxiety, and depression, were independent factors in infertility. The risk of infertility increased statistically significantly with the exposure time interval of OSA. Furthermore, the patients with OSA who did not receive treatment for OSA had an increased risk of infertility.

In reviews of the existing literature, the prevalence of OSA was reported to be 4% in middle-aged (30-60 years) men and 2% in middle-aged women.^21^ A higher prevalence in high-income countries (10% in women and 20% in men) was also reported.^9^ Obesity was found to be the most critical risk factor,^22^ and male sex was the second most important risk factor.^21^ Many studies have elucidated the association between OSA and male infertility, and an analysis of polysomnography using an apnea index has been conducted.^10^

Obstructive sleep apnea may play an important role in oxygen desaturation, hypercapnia, and sleep fragmentation, which were associated with cardiovascular, metabolic, and neurocognitive complications.^23^ Palnitkar et al^10^ proposed that OSA and other sleep disorders may lead to infertility through increasing oxidative stress, insulin resistance, systemic inflammation, and aberrant reproductive hormone secretion.^10^ Previous studies have suggested several explanatory mechanisms. Nocturnal hypoxemia-related changes in the hypothalamic-pituitary-gonadal axis and sleep fragmentation were among the suggested mechanisms.^24,25^ Several studies have also found an association between OSA and low testosterone levels.^11,12,13^ High levels of sperm DNA fragmentation were found to be a novel factor in male infertility,^26^ which has been associated with oxidative stress.^27^ The high oxidative stress may have a direct association with sperm quality and fertility in patients with OSA.^27^ In an animal model, Torres et al^28^ indicated that chronic, high-frequency, intermittent hypoxia mimicking OSA reduced male fertility and testicle antioxidant capacity in middle-aged mice. Moreover, increased levels of proinflammatory cytokines may be associated with spermatogenesis, sperm quality, and fertility.^29,30^ The escalation of inflammatory cytokines, including interleukin 6, tumor necrosis factor, and C-reactive protein, was reported in patients with OSA.^31^ Findings of the previous research and the present study provide evidence that OSA is a substantial risk factor in infertility. In addition, this study observed an association between increasing exposure time interval of OSA and increased risk of infertility.

Continuous positive airway pressure is reportedly the first choice of treatment for OSA, although surgical interventions such as UPPP may be advantageous for certain groups of patients.^32^ In this study, only approximately 50% of patients with OSA received treatment, and those who did not receive treatment had a markedly increased risk of infertility. The risk of infertility was increased without CPAP, surgical treatment, or both. However, previous studies on the treatment of OSA and reproductive hormones, such as testosterone and prolactin, had conflicting results.^14,15,32^ These studies had limited sample size and examined different CPAP treatment durations. Therefore, results of this study support the hypothesis that the treatment of OSA decreases the risk of infertility.

Obesity and metabolic syndrome have been recognized as risk factors of male infertility.^7,8^ In addition, OSA has been proposed as a modulating factor in obesity, metabolic syndrome, and infertility.^33^ Because obesity-related treatment is not reimbursed in Taiwan, the true incidence of obesity might be underestimated through records of *ICD-9-CM* codes. However, it was noteworthy that, in this research, obesity was an independent risk factor in infertility after the multivariate conditional logistic regression analysis. No single *ICD-9-CM* code for metabolic syndrome was found; therefore, the association between metabolic syndrome and infertility was not evaluated in this study.

This study showed that younger age (<40 years) was an independent risk factor in male infertility compared with age 45 years or older. In men with OSA, those younger than 40 years were more likely to become infertile. A previous study showed that young female patients had a higher risk of infertility because of higher stress levels.^20^ A higher prevalence of anxiety and depression was found in the younger age group than in the general population.^20^ Therefore, we applied the same model; however, we observed no statistically significant pattern in the prevalence of anxiety and depression between the age groups (eTable 2 in the Supplement). A possible reason for this pattern might be that younger male patients compared with older men pay more attention to issues of infertility and visit the outpatient andrology clinic more often. Thus, the higher prevalence of infertility in young patients might be associated with the *ICD-9-CM* diagnosis codes used in the NHIRD.

As for the use of a health insurance database for tracking the prevalence of disease diagnoses, all cases included in this study had long-term follow-up data, rather than short-term causality data, which are often used in cross-sectional studies. Therefore, this study may support physicians by further elucidating the association between OSA and male infertility.

### Limitations

This study has some limitations. First, the NHIRD did not provide laboratory data and comprehensive information, including blood pressure, body mass index, work shift, family history, smoking habit, alcohol consumption, and medications or treatments at the outpatient department. The lack of these data and information might be confounding factors, which could have affected the results of this study. Second, the NHIRD did not provide data on the parameters of male infertility, such as sperm motility, sperm counts, sperm DNA fragmentation, reproductive hormone levels, or genetic karyotyping. Third, the polysomnographic data were not available. We could not associate the disease severity with the occurrence of infertility or examine the association between the degree of hypoxemia or sleep fragmentation and infertility. Fourth, although the actual exposure time to OSA was unknown, long-term exposure to OSA was definitely associated with male infertility, according to the diagnosis codes in the NHIRD. Fifth, the outcome of treatment was not evaluated because it was difficult to assess the implication of the intervention for male infertility just by using NHIRD data. We analyzed only the CPAP and UPPP treatment modalities, which were the commonly used therapies for OSA in Taiwan. A further prospective study with comprehensive information is necessary to elucidate the association of OSA treatments with male infertility in patients with OSA.

## Conclusions

Sleep disturbance, including OSA, appeared to be associated with metabolic and neurocognitive functions, and this problem seems to be increasing in the modern era. Findings from this study appear to support the hypothesis that OSA increases the risk of infertility in male patients, and the risk is associated with the OSA exposure time interval. Moreover, OSA without management is associated with an increased risk of infertility. Early recognition of OSA and its interventions may decrease the risk of subsequent complications, including infertility.

## Notes

Supplement. **eTable 1.** The ICD-9-CM Codes **eTable 2.** Percentage of Anxiety and Depression in Different Age Groups Among the Study and the Control Group **eFigure 1.** The Flowchart of Study Design (Nested Case-Control Study) From National Health Insurance Research Database in Taiwan **eFigure 2.** Factors of Infertility Stratified by OSA and Obesity by Using Conditional Logistic Regression **eFigure 3.** Factors of Infertility Stratified by OSA and Cardiometabolic Disease by Using Conditional Logistic Regression
