# Errors in Table 3

**DOI:** 10.1001/jamanetworkopen.2022.24322

**Published:** 2022-07-12

In the Original Investigation titled “Association of Obstructive Sleep Apnea With the Risk of Male Infertility in Taiwan,” published on January 21, 2021, several of the percentages listed in the fourth column of Table 3 were incorrect. This article has been corrected.^1^
