# Supplementary material for: Association of Obstructive Sleep Apnea With the Risk of Male Infertility in Taiwan
Source: JAMA Netw Open. 2021 Jan 21;4(1):e2031846. doi: 10.1001/jamanetworkopen.2020.31846 (PMC7821032; doi:10.1001/jamanetworkopen.2020.31846)
Supplement: Supplement. — eTable 1. The ICD-9-CM Codes eTable 2. Percentage of Anxiety and Depression in Different Age Groups Among the Study and the Control Group eFigure 1. The Flowchart of Study Design (Nested Case-Control Study) From National Health Insurance Research Database in Taiwan eFigure 2. Factors of Infertility Stratified by OSA and Obesity by Using Conditional Logistic Regression eFigure 3. Factors of Infertility Stratified by OSA and Cardiometabolic Disease by Using Conditional Logistic Regression [file jamanetwopen-e2031846-s001.pdf]

Supplementary Online Content

Jhuang YH, Chung CH, Wang ID, et al. Association of obstructive sleep apnea with the risk of male infertility in Taiwan. *JAMA Netw Open*. 2021;4(1): e2031846.
doi:10.1001/jamanetworkopen.2020.31846

eTable 1. The ICD-9-CM Codes

eTable 2. Percentage of Anxiety and Depression in Different Age Groups Among the Study and the Control Group

eFigure 1. The Flowchart of Study Design (Nested Case-Control Study) From National Health Insurance Research Database in Taiwan

eFigure 2. Factors of Infertility Stratified by OSA and Obesity by Using Conditional Logistic Regression

eFigure 3. Factors of Infertility Stratified by OSA and Cardiometabolic Disease by Using Conditional Logistic Regression

This supplementary material has been provided by the authors to give readers additional information about their work.

©2021 Jhuang YH et al. JAMA Network Open

eTable 1. The ICD-9-CM Codes

**Diagnosis**	**ICD-9-CM codes**
Infertility	606
Obstructive sleep apnea	327.23, 780.51, 780.53, 780.57, 17008A-17008B
Polysomnography	17008A-17008B
Uvulopalatopharyngoplasty	66025B
Erectile dysfunction	607.84
early ejaculation	302.75
Genital warts	078.11
Chlamydia	078.88, 079.88, 079.98, 099.41, 099.53-099.55
Gonorrhea	098.0-098.34
Syphilis	091.0-091.2
Human immunodeficiency virus	042-044, 795.8, V08
Epididymitis / orchitis	098.13, 098.33, 604.0, 604.9
Mumps orchitis	072.0
Varicocele	465.4
Genital tuberculosis	016.50
Alcohol abuse / dependence syndrome	303, 305.0
Drug abuse / dependence	304.0-034.93, 305.2-305.93
Prolactinema	253.1
Hypogonadotropic hypogonadism	253.4
Cryptorchidism	752.5
Testicular hypofunction	257.2
Adrenogenital disorders	255.2
Thyroid disorder	
Hyperthyroidism	242.0-242.4, 242.8-242.9
Hypothyroidism	244.0-244.3, 244.8-244.9, 245.2
Drugs	
Alkylating agents	Cyclophosphamide, Chlorambucil, Busulfan
Platinum	Cisplatin, Carboplatin
Antiepileptic medications	Carbamazepine, Oxcarbazepine, Valproate sodium
Operations on the male genital organs	OP60-OP64
Hypertension	401-405
Diabetes mellitus	250
Hyperlipidemia	272

©2021 Jhuang YH et al. JAMA Network Open

Chronic obstructive pulmonary disease	490-496
Chronic kidney disease	580-589
Coronary heart disease	410-414
Congestive heart failure	428-429
Liver cirrhosis	571.2, 571.5-571.6, 572.2-575.4, 572.8, 573.0
Stroke	430-438
Cancer	140-238
Obesity	278
Epilepsy	345
Hepatitis B virus	070.20, 070.22, 070.30, 070.327, V02.61
Hepatitis C virus	070.41, 070.44, 070.51, 070.54, 070.7, V02.62
Anxiety	300.0, 300.2-300.3, 308.3, 309.81
Depression	296.2-296.3, 300.4, 311

©2021 Jhuang YH et al. JAMA Network Open

eTable 2. Percentage of Anxiety and Depression in Different Age Groups Among the Study and the Control Group

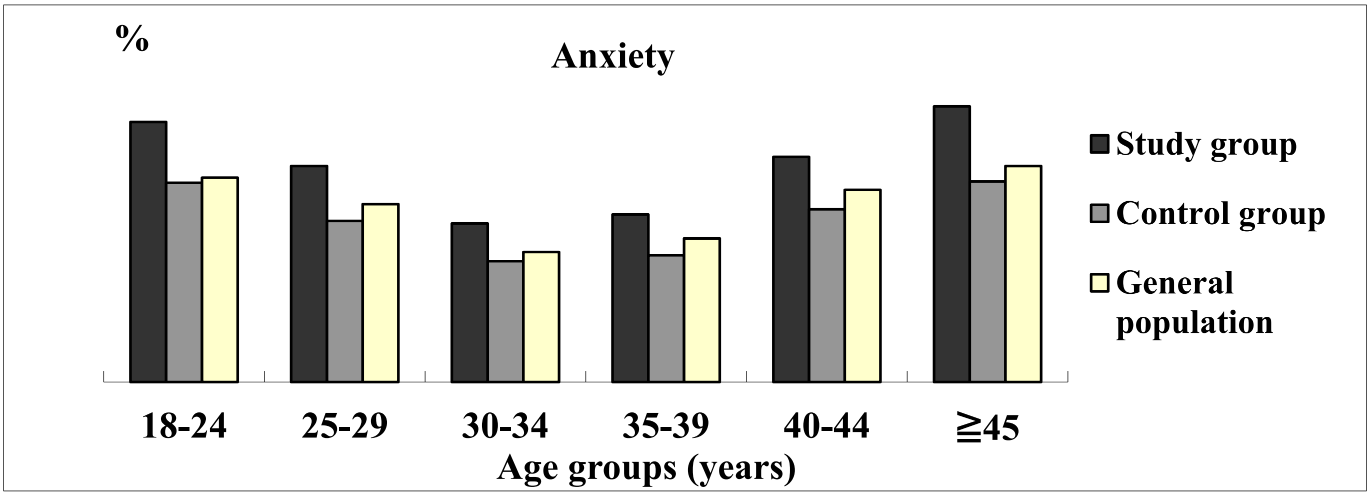

Anxiety (%)			
**Age group**	**Study group**	**Control group**	**General population**
**18-24**	4.02	3.08	3.16
**25-29**	3.34	2.49	2.75
**30-34**	2.45	1.87	2.01
**35-39**	2.59	1.96	2.22
**40-44**	3.48	2.67	2.97
**□ 45**	4.26	3.10	3.34

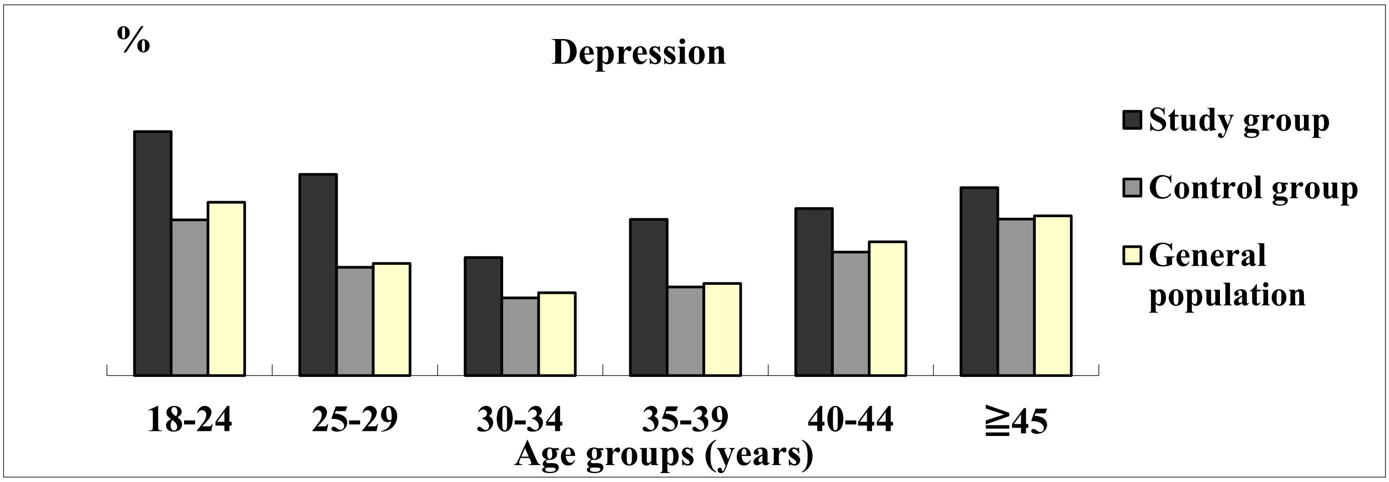

Depression (%)			
**Age group**	**Study group**	**Control group**	**General population**
**18-24**	6.22	3.97	4.42
**25-29**	5.13	2.76	2.86
**30-34**	3.01	1.98	2.11

©2021 Jhuang YH et al. JAMA Network Open

**35-39**	3.98	2.26	2.35
**40-44**	4.26	3.15	3.41
**□ 45**	4.79	3.99	4.07

©2021 Jhuang YH et al. JAMA Network Open

eFigure 1. The Flowchart of Study Design (Nested Case-Control Study) From National Health Insurance Research Database in Taiwan

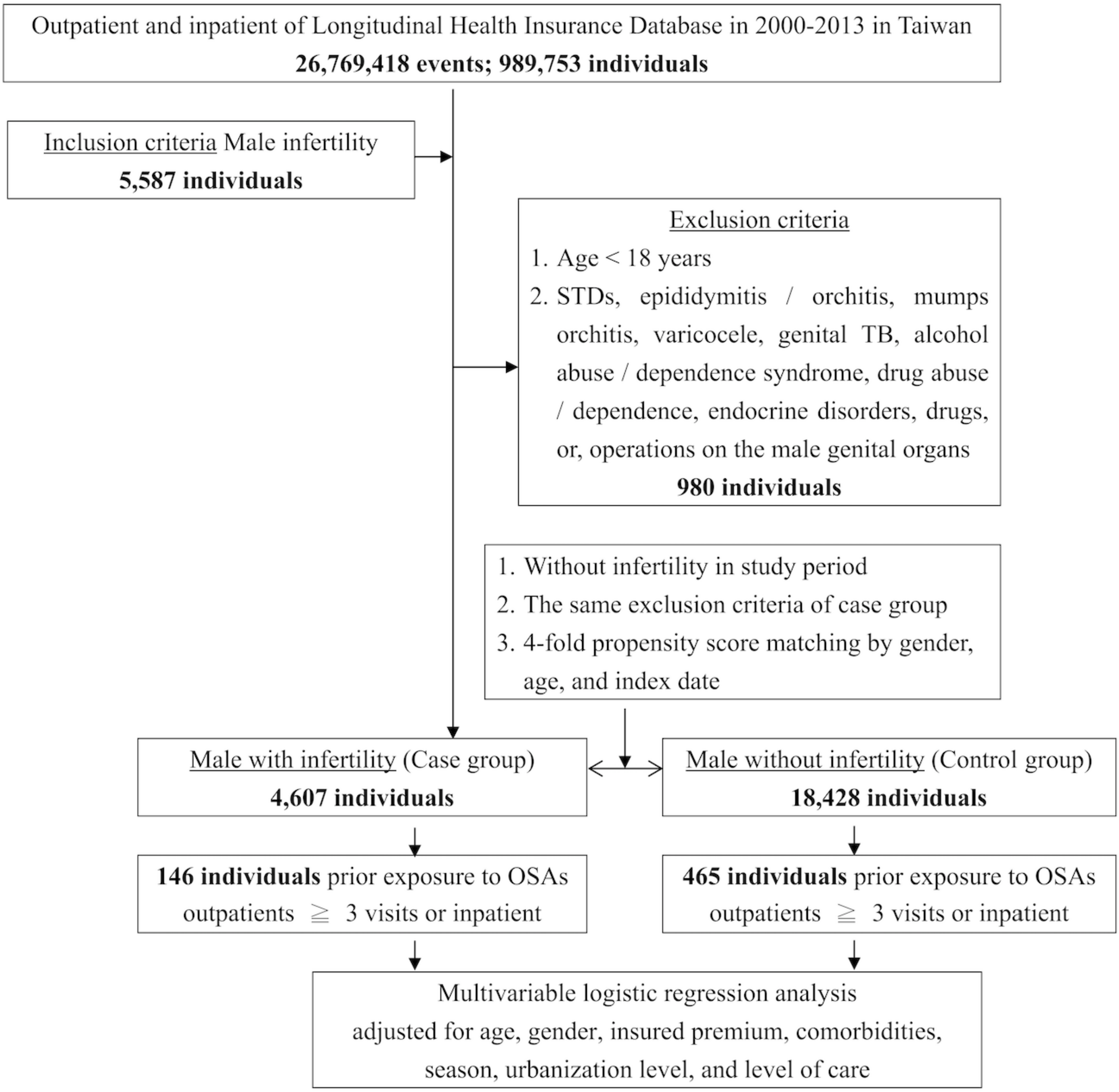

©2021 Jhuang YH et al. JAMA Network Open

eFigure 2. Factors of Infertility Stratified by OSA and Obesity by Using Conditional Logistic Regression

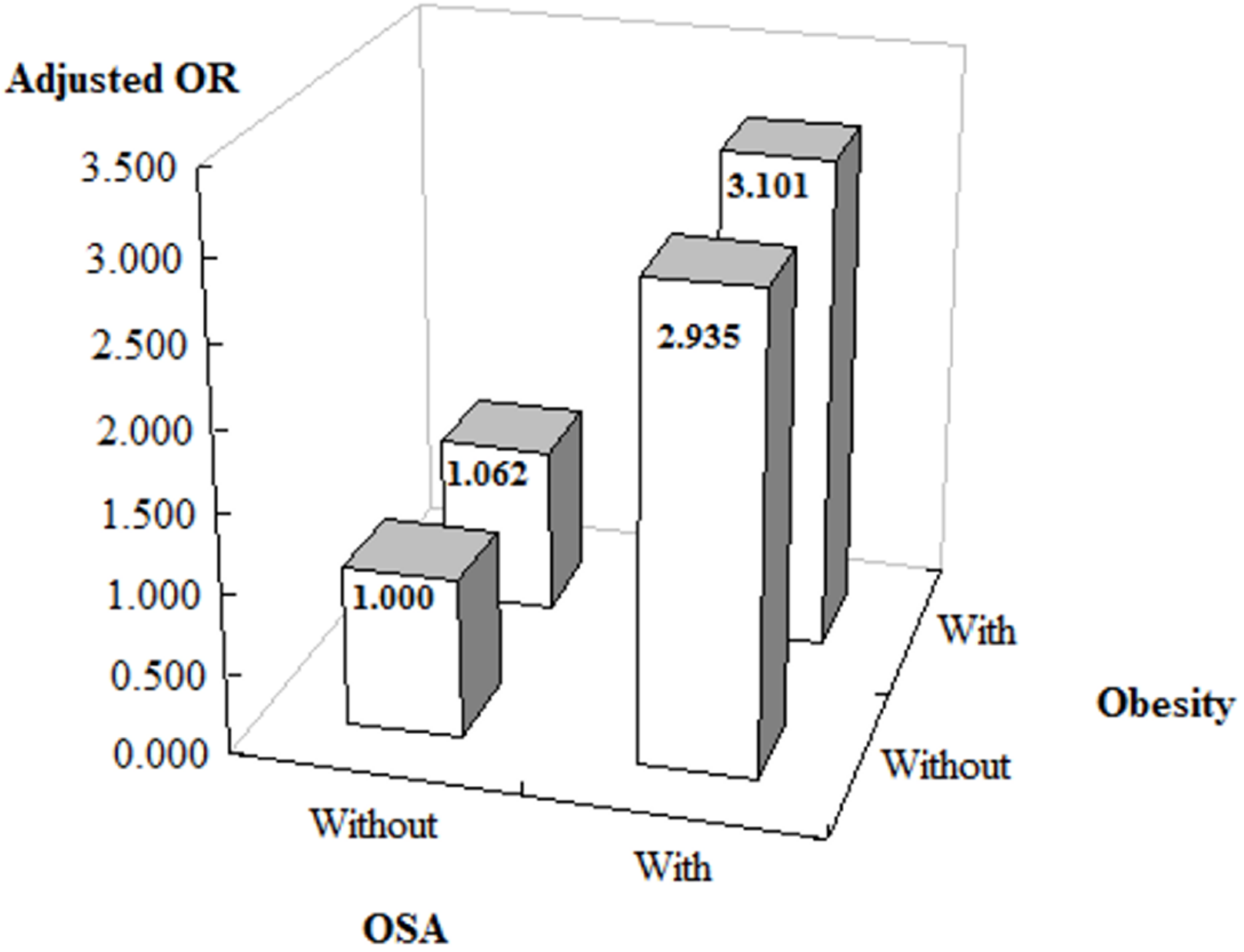

OSA	Obesity	Adjusted OR	95% CI	95% CI	*P*
Without	Without	Reference			
Without	With	1.062	0.946	1.435	0.274
With	Without	2.935	1.204	6.114	<0.001
With	With	2.997	2.015	6.283	<0.001

OSA: obstructive sleep apnea; Adjusted OR (odds ratio): Adjusted variables listed in Table 3. ; CI = confidence interval

©2021 Jhuang YH et al. JAMA Network Open

eFigure 3. Factors of Infertility Stratified by OSA and Cardiometabolic Disease by Using Conditional Logistic Regression

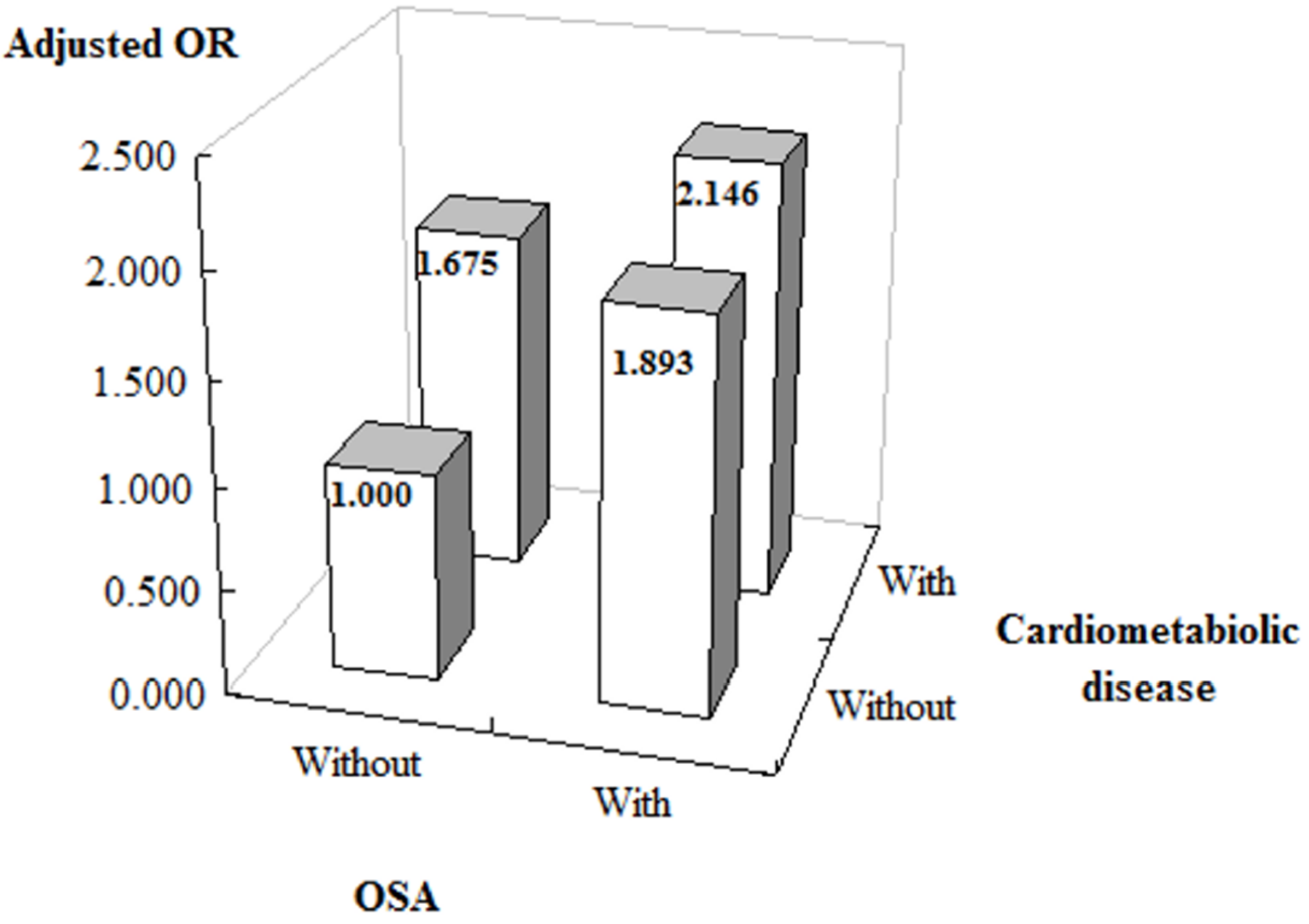

OSA	Cardiometabiolic disease	Adjusted OR	95% CI	95% CI	*P*
Without	Without	Reference			
Without	With	1.675	1.082	2.575	0.001
With	Without	1.893	1.304	2.893	<0.001
With	With	2.146	1.859	3.260	<0.001

OSA: obstructive sleep apnea; Cardiometabiolic disease: Hypertension / Diabetes mellitus / Hyperlipidemia; Adjusted OR (odds ratio): Adjusted variables listed in Table 3. ; CI = confidence interval

©2021 Jhuang YH et al. JAMA Network Open
